# Supplementary figures and images for: Evolution of global development cooperation: An analysis of aid flows with hierarchical stochastic block models
Source: PLoS One. 2022 Aug 3;17(8):e0272440. doi: 10.1371/journal.pone.0272440 (PMC9348651; doi:10.1371/journal.pone.0272440)

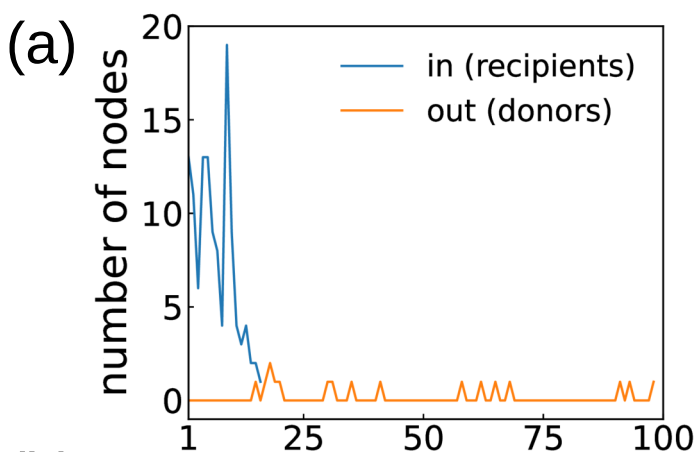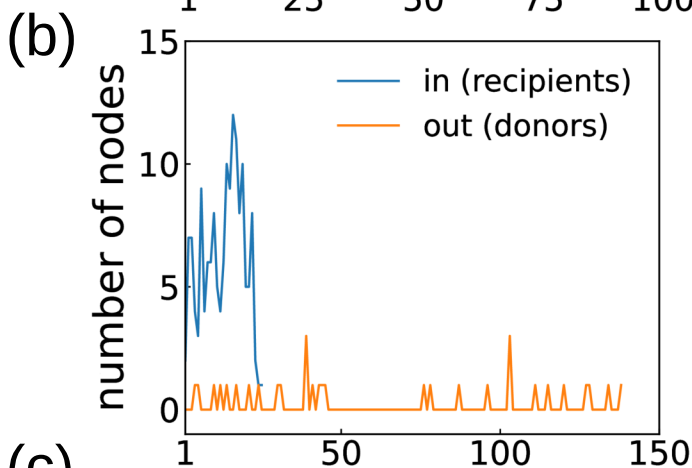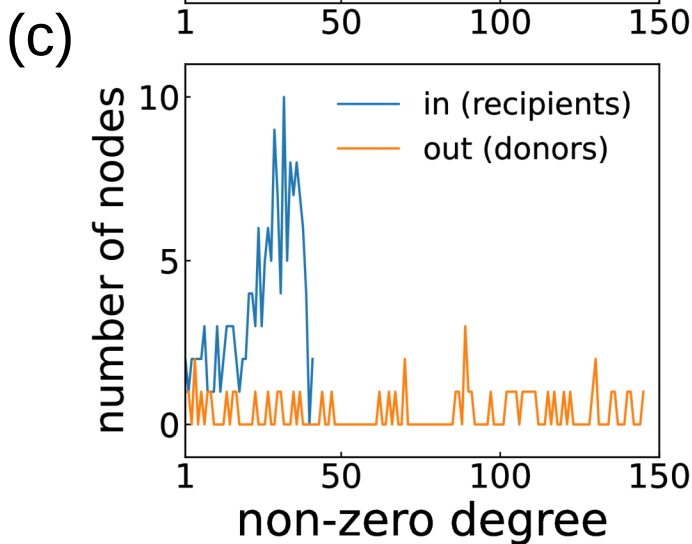

Supplement: S1 Fig — (PDF) [file pone.0272440.s001.pdf]

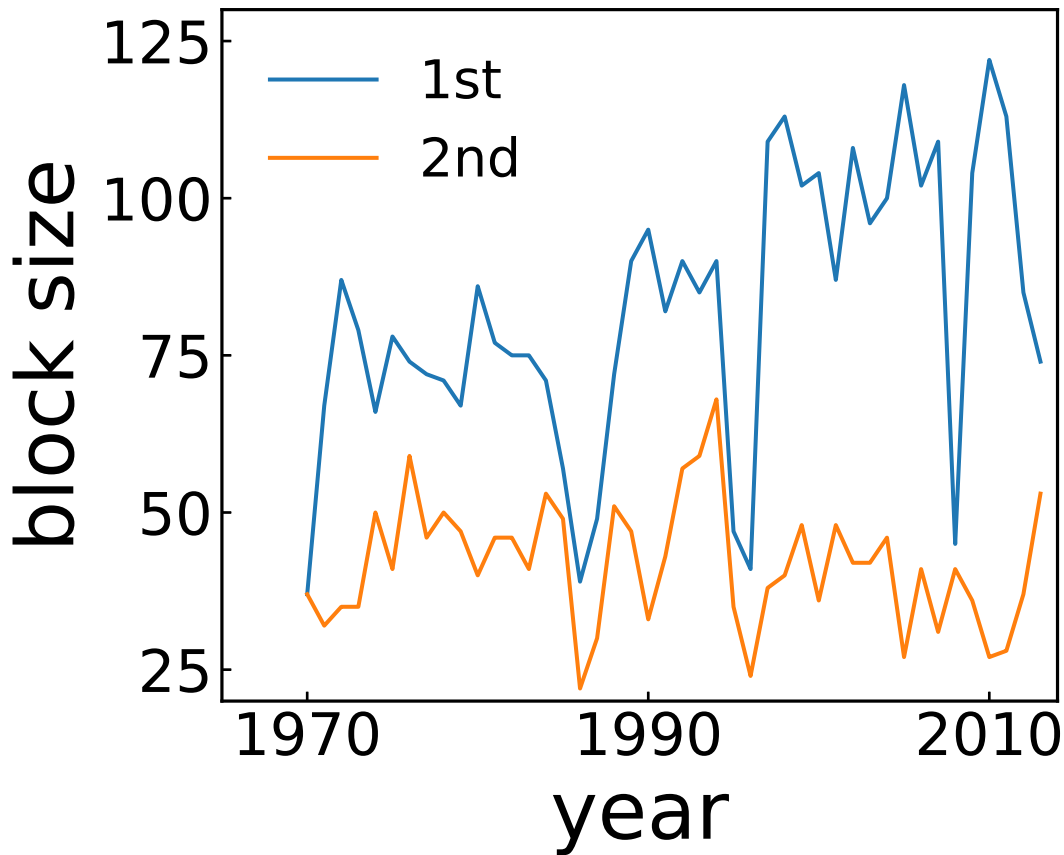

Supplement: S2 Fig — (PDF) [file pone.0272440.s002.pdf]
