# Supplementary material for: Evolution of global development cooperation: An analysis of aid flows with hierarchical stochastic block models
Source: PLoS One. 2022 Aug 3;17(8):e0272440. doi: 10.1371/journal.pone.0272440 (PMC9348651; doi:10.1371/journal.pone.0272440)
Supplement: S1 Table — (PDF) [file pone.0272440.s003.pdf]

Table S1. List of actors in stable blocks in 1970.

block ID	actors
0	Afghanistan, Algeria, Botswana, Brazil, Burkina Faso, Burundi, Cambodia, Cameroon, Central African Republic, Congo, Democratic Republic of the Congo, Ethiopia, Gabon, Ghana, India, Indonesia, Kenya, Korea, Lao People's Democratic Republic, Madagascar, Mali, Morocco, Niger, Nigeria, Pakistan, Peru, Philippines, Rwanda, Senegal, Sri Lanka, Tanzania, Thailand, Tunisia, Turkey, Uganda, Viet Nam, Zambia
1	Australia, Benin, Bolivia, Chad, Chile, Chinese Taipei, Colombia, Costa Rica, Cote d'Ivoire, Cyprus, Dominican Republic, Ecuador, Egypt, El Salvador, Eswatini, Fiji, Gambia, Guatemala, Guinea, Guyana, Honduras, Hong Kong (China), Iran, Israel, Jordan, Lebanon, Lesotho, Liberia, Malawi, Malaysia, Malta, Mauritania, Mauritius, Mexico, Myanmar, Nepal, New Caledonia, Nicaragua, Panama, Papua New Guinea, Sierra Leone, Singapore, Somalia, Sudan, Togo, Uruguay, Vanuatu, Venezuela
2	Angola, Argentina, Bahamas, Bahrain, Barbados, Belize, Bermuda, Bhutan, Brunei Darussalam, Comoros, Cuba, Djibouti, French Polynesia, Gibraltar, Haiti, Iraq, Jamaica, Kiribati, Libya, Maldives, Mozambique, Netherlands Antilles, Northern Mariana Islands, Paraguay, Saint Helena, Samoa, Saudi Arabia, Seychelles, Solomon Islands, Suriname, Syrian Arab Republic, Tonga, Trinidad and Tobago, United Arab Emirates, Wallis and Futuna, Yemen, Zimbabwe
3	Austria, Denmark, EU Institutions, France, International Development Association [IDA], Japan, Netherlands, Norway, Sweden
4	Belgium, Canada, Germany, Italy, Switzerland, United Kingdom, United States
