# Supplementary material for: Evolution of global development cooperation: An analysis of aid flows with hierarchical stochastic block models
Source: PLoS One. 2022 Aug 3;17(8):e0272440. doi: 10.1371/journal.pone.0272440 (PMC9348651; doi:10.1371/journal.pone.0272440)
Supplement: S2 Table — (PDF) [file pone.0272440.s004.pdf]

Table S2. List of actors in stable blocks in 1990.

block ID	actors
0	Afghanistan, Algeria, Angola, Argentina, Bangladesh, Benin, Bhutan, Bolivia, Botswana, Brazil, Burkina Faso, Burundi, Cabo Verde, Cambodia, Cameroon, Central African Republic, Chad, Chile, China (People's Republic of), Colombia, Comoros, Congo, Costa Rica, Cote d'Ivoire, Cuba, Democratic Republic of the Congo, Djibouti, Dominican Republic, Ecuador, Egypt, El Salvador, Equatorial Guinea, Eswatini, Ethiopia, Gabon, Gambia, Ghana, Guatemala, Guinea, Guinea-Bissau, Guyana, Haiti, Honduras, India, Indonesia, Iran, Jamaica, Jordan, Kenya, Lao People's Democratic Republic, Lebanon, Lesotho, Liberia, Madagascar, Malawi, Malaysia, Maldives, Mali, Mauritania, Mauritius, Mexico, Morocco, Mozambique, Namibia, Nepal, Nicaragua, Niger, Nigeria, Pakistan, Papua New Guinea, Paraguay, Peru, Philippines, Rwanda, Saint Lucia, Samoa, Sao Tome and Principe, Senegal, Seychelles, Sierra Leone, Somalia, Sri Lanka, Sudan, Tanzania, Thailand, Togo, Tonga, Tunisia, Turkey, Uganda, Uruguay, Viet Nam, Yemen, Zambia, Zimbabwe
1	Albania, Anguilla, Antigua and Barbuda, Aruba, Bahamas, Bahrain, Barbados, Belize, Bermuda, British Virgin Islands, Brunei Darussalam, Cayman Islands, Chinese Taipei, Cook Islands, Cyprus, Democratic People's Republic of Korea, Dominica, French Polynesia, Gibraltar, Grenada, Iraq, Kiribati, Libya, Macau (China), Mayotte, Mongolia, Montserrat, Nauru, Netherlands Antilles, New Caledonia, Niue, Oman, Qatar, Saint Helena, Saint Kitts and Nevis, Saint Vincent and the Grenadines, Saudi Arabia, Solomon Islands, Suriname, Tokelau, Trinidad and Tobago, Turks and Caicos Islands, Tuvalu, United Arab Emirates, Vanuatu, Wallis and Futuna
2	Australia, Austria, Belgium, Canada, EU Institutions, Finland, France, Germany, Italy, Japan, Netherlands, Switzerland, United Kingdom, United States
3	Fiji, Hong Kong (China), Israel, Malta, Myanmar, Northern Mariana Islands, Panama, Singapore, Syrian Arab Republic, Venezuela
4	African Development Fund [AfDF], Denmark, IFAD, International Development Association [IDA], Ireland, Norway, Sweden
5	Arab Bank for Economic Development in Africa [BADEA], Arab Fund (AFESD), Caribbean Development Bank [CarDB], Islamic Development Bank [IsDB], New Zealand, Nordic Development Fund [NDF], OPEC Fund for International Development [OPEC Fund]
6	Korea, Kuwait
