# Supplementary material for: Evolution of global development cooperation: An analysis of aid flows with hierarchical stochastic block models
Source: PLoS One. 2022 Aug 3;17(8):e0272440. doi: 10.1371/journal.pone.0272440 (PMC9348651; doi:10.1371/journal.pone.0272440)
Supplement: S3 Table — (PDF) [file pone.0272440.s005.pdf]

Table S3. List of actors in stable blocks in 2010.

block ID	actors
0	Afghanistan, Albania, Algeria, Angola, Argentina, Armenia, Azerbaijan, Bangladesh, Belarus, Benin, Bolivia, Bosnia and Herzegovina, Botswana, Brazil, Burkina Faso, Burundi, Cambodia, Cameroon, Central African Republic, Chad, Chile, Colombia, Congo, Costa Rica, Cote d'Ivoire, Croatia, Cuba, Democratic Republic of the Congo, Djibouti, Dominican Republic, Ecuador, Egypt, El Salvador, Eswatini, Ethiopia, Gambia, Georgia, Ghana, Guatemala, Guinea, Guinea-Bissau, Haiti, Honduras, India, Indonesia, Iran, Iraq, Jordan, Kazakhstan, Kenya, Kosovo, Kyrgyzstan, Lao People's Democratic Republic, Lebanon, Lesotho, Liberia, Madagascar, Malawi, Malaysia, Mali, Mauritania, Mexico, Moldova, Mongolia, Montenegro, Morocco, Mozambique, Myanmar, Namibia, Nepal, Nicaragua, Niger, Nigeria, North Macedonia, Pakistan, Palestinian Adm. Areas, Panama, Paraguay, Peru, Philippines, Rwanda, Senegal, Serbia, Sierra Leone, Somalia, South Africa, Sri Lanka, Sudan, Syrian Arab Republic, Tajikistan, Tanzania, Thailand, Togo, Tunisia, Turkey, Turkmenistan, Uganda, Ukraine, Uruguay, Uzbekistan, Venezuela, Viet Nam, Yemen, Zambia, Zimbabwe
1	Anguilla, Antigua and Barbuda, Barbados, Belize, Cook Islands, Dominica, Equatorial Guinea, Fiji, Grenada, Guyana, Jamaica, Kiribati, Marshall Islands, Mauritius, Mayotte, Micronesia, Montserrat, Nauru, Niue, Oman, Palau, Saint Helena, Saint Kitts and Nevis, Saint Lucia, Saint Vincent and the Grenadines, Samoa, Sao Tome and Principe, Seychelles, Solomon Islands, Suriname, Tokelau, Tonga, Trinidad and Tobago, Tuvalu, Vanuatu, Wallis and Futuna
2	Bhutan, Cabo Verde, Canada, Comoros, Democratic People's Republic of Korea, Eritrea, Finland, France, Gabon, Ireland, Korea, Libya, Maldives, Papua New Guinea, Spain, Timor-Leste, UN Peacebuilding Fund [UNPBF], United Kingdom
3	Austria, Belgium, Bill & Melinda Gates Foundation, Denmark, Greece, Italy, Luxembourg, Netherlands, Norway, Sweden, Switzerland
4	EU Institutions, Global Alliance for Vaccines and Immunization [GAVI], Global Environment Facility [GEF], Global Fund, International Development Association [IDA], UNAIDS, UNDP, UNFPA, UNICEF
5	Adaptation Fund, African Development Bank [AfDB], Arab Fund (AFESD), Council of Europe Development Bank [CEB], Nordic Development Fund [NDF], OSCE, UNECE, UNRWA
6	African Development Fund [AfDF], Arab Bank for Economic Development in Africa [BADEA], Caribbean Development Bank [CarDB], IFAD, IMF (Concessional Trust Funds), Islamic Development Bank [IsDB], Kuwait, OPEC Fund for International Development [OPEC Fund]
7	Australia, New Zealand, Portugal, United Arab Emirates
8	Germany, Japan, United States
9	China (People's Republic of)
