# Supplementary material for: Evolution of global development cooperation: An analysis of aid flows with hierarchical stochastic block models
Source: PLoS One. 2022 Aug 3;17(8):e0272440. doi: 10.1371/journal.pone.0272440 (PMC9348651; doi:10.1371/journal.pone.0272440)
Supplement: S4 Table — (PDF) [file pone.0272440.s006.pdf]

Table S4. List of actors in the sample block structure in 1970 in Fig. 2

block ID	actors
0	Angola, Argentina, Bahamas, Bahrain, Barbados, Belize, Benin, Bermuda, Bhutan, Brunei Darussalam, Chile, Chinese Taipei, Colombia, Comoros, Costa Rica, Cuba, Cyprus, Djibouti, Dominican Republic, El Salvador, Fiji, French Polynesia, Gibraltar, Guinea, Guyana, Haiti, Honduras, Hong Kong (China), Iraq, Israel, Jamaica, Jordan, Kiribati, Lebanon, Liberia, Libya, Maldives, Malta, Mauritania, Mozambique, Netherlands Antilles, New Caledonia, Nicaragua, Northern Mariana Islands, Panama, Paraguay, Saint Helena, Samoa, Saudi Arabia, Seychelles, Singapore, Solomon Islands, Somalia, Sudan, Suriname, Syrian Arab Republic, Tonga, Trinidad and Tobago, United Arab Emirates, Uruguay, Vanuatu, Venezuela, Wallis and Futuna, Yemen, Zimbabwe
1	Afghanistan, Algeria, Bolivia, Botswana, Brazil, Burkina Faso, Burundi, Cambodia, Cameroon, Central African Republic, Chad, Congo, Cote d'Ivoire, Democratic Republic of the Congo, Ecuador, Egypt, Eswatini, Ethiopia, Gabon, Gambia, Ghana, Guatemala, India, Indonesia, Iran, Kenya, Korea, Lao People's Democratic Republic, Lesotho, Madagascar, Malawi, Malaysia, Mali, Mauritius, Mexico, Morocco, Myanmar, Nepal, Niger, Nigeria, Pakistan, Papua New Guinea, Peru, Philippines, Rwanda, Senegal, Sierra Leone, Sri Lanka, Tanzania, Thailand, Togo, Tunisia, Turkey, Uganda, Viet Nam, Zambia
2	Australia, Austria, Denmark, EU Institutions, France, International Development Association [IDA], Japan, Netherlands, Norway, Sweden
3	Belgium, Canada, Germany, Italy, Switzerland, United Kingdom, United States
