# Supplementary material for: Evolution of global development cooperation: An analysis of aid flows with hierarchical stochastic block models
Source: PLoS One. 2022 Aug 3;17(8):e0272440. doi: 10.1371/journal.pone.0272440 (PMC9348651; doi:10.1371/journal.pone.0272440)
Supplement: S5 Table — (PDF) [file pone.0272440.s007.pdf]

Table S5. List of actors in the sample block structure in 1990 in Fig. 2

block ID	actors
0	Afghanistan, Algeria, Angola, Argentina, Bahrain, Bangladesh, Benin, Bhutan, Bolivia, Botswana, Brazil, Brunei Darussalam, Burkina Faso, Burundi, Cabo Verde, Cambodia, Cameroon, Central African Republic, Chad, Chile, China (People's Republic of), Chinese Taipei, Colombia, Comoros, Congo, Costa Rica, Cote d'Ivoire, Cuba, Democratic Republic of the Congo, Djibouti, Dominican Republic, Ecuador, Egypt, El Salvador, Equatorial Guinea, Eswatini, Ethiopia, Fiji, Gabon, Gambia, Ghana, Grenada, Guatemala, Guinea, Guinea-Bissau, Guyana, Haiti, Honduras, Hong Kong (China), India, Indonesia, Iran, Israel, Jamaica, Jordan, Kenya, Lao People's Democratic Republic, Lebanon, Lesotho, Liberia, Libya, Madagascar, Malawi, Malaysia, Maldives, Mali, Malta, Mauritania, Mauritius, Mexico, Mongolia, Morocco, Mozambique, Myanmar, Namibia, Nepal, Nicaragua, Niger, Nigeria, Northern Mariana Islands, Oman, Pakistan, Panama, Papua New Guinea, Paraguay, Peru, Philippines, Rwanda, Saint Lucia, Samoa, Sao Tome and Principe, Saudi Arabia, Senegal, Seychelles, Sierra Leone, Singapore, Somalia, Sri Lanka, Sudan, Syrian Arab Republic, Tanzania, Thailand, Togo, Tonga, Tunisia, Turkey, Uganda, Uruguay, Venezuela, Viet Nam, Yemen, Zambia, Zimbabwe
1	Albania, Anguilla, Antigua and Barbuda, Aruba, Bahamas, Barbados, Belize, Bermuda, British Virgin Islands, Cayman Islands, Cook Islands, Cyprus, Democratic People's Republic of Korea, Dominica, French Polynesia, Gibraltar, Iraq, Kiribati, Macau (China), Mayotte, Montserrat, Nauru, Netherlands Antilles, New Caledonia, Niue, Qatar, Saint Helena, Saint Kitts and Nevis, Saint Vincent and the Grenadines, Solomon Islands, Suriname, Tokelau, Trinidad and Tobago, Turks and Caicos Islands, Tuvalu, United Arab Emirates, Vanuatu, Wallis and Futuna
2	Australia, Austria, Belgium, Canada, EU Institutions, Finland, France, Germany, Italy, Japan, Netherlands, Switzerland, United Kingdom, United States
3	African Development Fund [AfDF], Denmark, IFAD, International Development Association [IDA], Ireland, Norway, OPEC Fund for International Development [OPEC Fund], Sweden
4	Arab Bank for Economic Development in Africa [BADEA], Arab Fund (AFESD), Caribbean Development Bank [CarDB], Islamic Development Bank [IsDB], New Zealand, Nordic Development Fund [NDF]
5	Korea, Kuwait
