# Supplementary material for: Evolution of global development cooperation: An analysis of aid flows with hierarchical stochastic block models
Source: PLoS One. 2022 Aug 3;17(8):e0272440. doi: 10.1371/journal.pone.0272440 (PMC9348651; doi:10.1371/journal.pone.0272440)
Supplement: S6 Table — (PDF) [file pone.0272440.s008.pdf]

Table S6. List of actors in the sample block structure in 2010 in Fig. 2

block ID	actors
0	Afghanistan, Albania, Algeria, Angola, Argentina, Armenia, Azerbaijan, Bangladesh, Belarus, Belize, Benin, Bhutan, Bolivia, Bosnia and Herzegovina, Botswana, Brazil, Burkina Faso, Burundi, Cabo Verde, Cambodia, Cameroon, Central African Republic, Chad, Chile, Colombia, Comoros, Congo, Costa Rica, Cote d'Ivoire, Croatia, Cuba, Democratic People's Republic of Korea, Democratic Republic of the Congo, Djibouti, Dominican Republic, Ecuador, Egypt, El Salvador, Eritrea, Eswatini, Ethiopia, Gabon, Gambia, Georgia, Ghana, Grenada, Guatemala, Guinea, Guinea-Bissau, Guyana, Haiti, Honduras, India, Indonesia, Iran, Iraq, Jamaica, Jordan, Kazakhstan, Kenya, Kosovo, Kyrgyzstan, Lao People's Democratic Republic, Lebanon, Lesotho, Liberia, Libya, Madagascar, Malawi, Malaysia, Maldives, Mali, Mauritania, Mauritius, Mexico, Moldova, Mongolia, Montenegro, Morocco, Mozambique, Myanmar, Namibia, Nepal, Nicaragua, Niger, Nigeria, North Macedonia, Pakistan, Palestinian Adm. Areas, Panama, Papua New Guinea, Paraguay, Peru, Philippines, Rwanda, Sao Tome and Principe, Senegal, Serbia, Seychelles, Sierra Leone, Solomon Islands, Somalia, South Africa, Sri Lanka, Sudan, Syrian Arab Republic, Tajikistan, Tanzania, Thailand, Timor-Leste, Togo, Tunisia, Turkey, Turkmenistan, Uganda, Ukraine, Uruguay, Uzbekistan, Venezuela, Viet Nam, Yemen, Zambia, Zimbabwe
1	Anguilla, Antigua and Barbuda, Barbados, Cook Islands, Dominica, Equatorial Guinea, Fiji, Kiribati, Marshall Islands, Mayotte, Micronesia, Montserrat, Nauru, Niue, Oman, Palau, Saint Helena, Saint Kitts and Nevis, Saint Lucia, Saint Vincent and the Grenadines, Samoa, Suriname, Tokelau, Tonga, Trinidad and Tobago, Tuvalu, Vanuatu, Wallis and Futuna
2	Austria, Belgium, Bill & Melinda Gates Foundation, Canada, Denmark, Greece, Ireland, Italy, Luxembourg, Netherlands, Norway, Sweden, Switzerland
3	Adaptation Fund, African Development Bank [AfDB], Arab Fund (AFESD), Caribbean Development Bank [CarDB], Council of Europe Development Bank [CEB], Nordic Development Fund [NDF], OSCE, UN Peacebuilding Fund [UNPBF], UNECE, UNRWA
4	EU Institutions, Global Alliance for Vaccines and Immunization [GAVI], Global Environment Facility [GEF], Global Fund, International Development Association [IDA], UNAIDS, UNDP, UNFPA, UNICEF
5	Finland, France, Germany, Japan, Korea, Spain, United Kingdom, United States
6	African Development Fund [AfDF], Arab Bank for Economic Development in Africa [BADEA], IFAD, IMF (Concessional Trust Funds), Islamic Development Bank [IsDB], Kuwait, OPEC Fund for International Development [OPEC Fund]
7	Australia, New Zealand, Portugal, United Arab Emirates
8	China (People's Republic of)
