# Supplementary material for: Evolution of global development cooperation: An analysis of aid flows with hierarchical stochastic block models
Source: PLoS One. 2022 Aug 3;17(8):e0272440. doi: 10.1371/journal.pone.0272440 (PMC9348651; doi:10.1371/journal.pone.0272440)
Supplement: S7 Table — (PDF) [file pone.0272440.s009.pdf]

Table : S7. Top 10 nodes for betweenness centrality

Rank	1970	1990	2010
1	United Kingdom	Japan	Japan
2	Germany	United Kingdom	France
3	United States	France	Germany
4	Italy	EU Institutions	United States
5	Belgium	Canada	United Kingdom
6	Canada	Netherlands	UNDP
7	Switzerland	Belgium	China (People's Republic of)
8	France	United States	Korea
9	EU Institutions	Austria	EU Institutions
10	Australia	Australia	Australia
